# A contingency perspective of pro-organizational motives, unethical pro-organizational behavior, and organizational citizenship behavior

**DOI:** 10.3389/fpsyg.2022.935210

**Published:** 2022-08-29

**Authors:** Ken Cheng, Panpan Hu, Limin Guo, Yifei Wang, Yinghui Lin

**Affiliations:** ^1^School of Management, Zhejiang University of Technology, Hangzhou, China; ^2^School of Economics and Management, Tongji University, Shanghai, China; ^3^School of Management, Shanghai University, Shanghai, China

**Keywords:** pro-organizational motives, unethical pro-organizational behavior, organizational citizenship behavior, moral identity, impression management motives

## Abstract

Although the effects of pro-organizational motives on pro-organizational behaviors [i.e., unethical pro-organizational behavior (UPB) and organizational citizenship behavior (OCB)] and their boundaries have been explored to some extent, extant studies are rather piecemeal and in need of synthesis and extension. Based on prior motivational research on pro-organizational behaviors, we developed a comprehensive contingent model in which moral identity and impression management motives would moderate the links between pro-organizational motives, UPB, and OCB. Adopting a time-lagged design, we collected data from 218 salespeople in an internet technology service company in China. Results showed that pro-organizational motives were positively related to UPB and OCB. Moral identity weakened the impact of pro-organizational motives on UPB but strengthened the influence of pro-organizational motives on OCB. Furthermore, we found that impression management motives strengthened the effects of pro-organizational motives on UPB and OCB, and the interaction of impression management motives and pro-organizational motives was stronger on UPB than on OCB. Theoretical and practical implications, limitations, and future directions are discussed.

## Introduction

Unethical pro-organizational behavior (UPB), a behavioral phenomenon that arouses academia’s great concern in the last decade (Mishra et al., 2022), refers to “actions that are intended to promote the effective functioning of the organization or its members (e.g., leaders) and violate core societal values, mores, laws, or standards of proper conduct” (Umphress and Bingham, 2011, p. 622). UPB is not only a kind of unethical behavior but also belongs to the category of pro-organizational behavior (Umphress et al., 2010). Pro-organizational behavior is an umbrella term, which includes UPB and ethical pro-organizational behavior. One very typical example of ethical pro-organizational behavior is organizational citizenship behavior (OCB). OCB is “individual behavior that is discretionary, not directly or explicitly recognized by the formal reward system, and that in the aggregate promotes the effective functioning of the organization” (Organ, 1988, p. 4). It is worth noting that UPB and OCB can coexist in the real world. For instance, it often occurs that a salesperson not only exaggerates the truth about his or her company’s products to clients but also defends his or her company when others criticize it. Extant research has found that organizational identification and positive social exchange in organizations can promote both UPB (e.g., Chen et al., 2016; Wang et al., 2019) and OCB (e.g., Moorman et al., 1998; Van Dick et al., 2006), suggesting that the pro-organizational motives lying beneath these factors may be the essential and common antecedents of UPB and OCB.

Although UPB and OCB are pro-organizational behaviors, their impacts on the organization are rather different. Extant research has found that UPB damages organizational performance (Baker et al., 2019), whereas OCB promotes organizational performance (Podsakoff et al., 2000). Considering the opposite influences of UPB and OCB on organizational performance and the positive effects of pro-organizational motives on UPB and OCB, it is of necessity and importance to identify the boundary conditions under which pro-organizational motives may lead to relatively good behavioral outcomes (e.g., less UPB and more OCB). So far, there are some studies directly or indirectly investigating the boundaries of the relationships between pro-organizational motives and pro-organizational behaviors, but their focuses are either on the boundaries of the relationship between pro-organizational motives and UPB (e.g., Matherne and Litchfield, 2012) or on the boundaries of the relationship between pro-organizational motives and OCB (e.g., Grant and Mayer, 2009). Meanwhile, it remains rather unclear whether the boundaries of the relationship between pro-organizational motives and OCB will also moderate the relationship between pro-organizational motives and UPB (and vice versa). We deem that it is necessary to fill these gaps. Because if the moderators that strengthen the link between pro-organizational motives and OCB can also amplify the positive effect of pro-organizational motives on UPB, managers should be cautious about these double-edged moderators. In sum, extant research has enriched our understanding of the boundaries of the relationship between pro-organizational motives and pro-organizational behaviors to some extent but still remains not enough.

To better answer the comprehensive question, when pro-organizational motives may result in relatively good behavioral outcomes, building an integrated model is highly needed. In the field of decision science, it is a near-universal consensus that decisions should be assessed by how good or bad the expected outcome is (Bennis et al., 2010). Conforming to this consensus, many studies have found that individuals’ attitudes toward the expected behavioral outcomes can interact to influence their behavioral decisions (e.g., Grant and Mayer, 2009; Takeuchi et al., 2015). For instance, Grant and Mayer (2009) argued that impression management motives can strengthen the effect of prosocial motives on affiliative citizenship behavior. Specifically, engaging in affiliative citizenship behavior can not only improve others’ welfare but also help actors establish a favorable image in the eyes of others. These expected outcomes are attractive for individuals who have high prosocial motives and impression management motives. Thus, impression management motives may guide employees with prosocial motives to engage in more affiliative citizenship behavior. Following this research stream, we proposed that the impacts of pro-organizational motives on UPB and OCB may be moderated by individuals’ attitudes toward the expected outcomes of UPB and OCB. In general, factors that can shape these attitudes may also likely function as boundary conditions (i.e., moderators). To concretize this idea, we need to analyze UPB and OCB and their potential outcomes.

On the one hand, although both UPB and OCB are pro-organizational behaviors, the former is unethical, whereas the latter is ethical. Therefore, for individuals who place greater importance on their moral self-image, UPB will be less attractive than OCB. In this vein, we infer that moral identity, a moral trait that reflects the extent to which one’s self-concept incorporates the importance of being a moral person (Aquino and Reed, 2002), may guide employees with pro-organizational motives to engage in more OCB and less UPB. On the other hand, although UPB damages organizational long-term interests, it may bring some short-term benefits to the organization (Wang et al., 2020), such as an increase in sales. Thus, UPB may, in turn, benefit the actor (Umphress et al., 2010), such as the establishment of a “good employee” image. Given that OCB can also improve one’s social image (Rioux and Penner, 2001), UPB and OCB may become welcome for individuals who want to sustain a positive image. In this vein, we infer that impression management motives, one’s desire to be seen positively and to avoid being seen negatively (Rioux and Penner, 2001), may guide employees with pro-organizational motives to conduct more UPB and OCB. Moreover, given that unethical behavior allows an employee to contribute to his or her organization beyond what can be achieved *via* moral means (Thau et al., 2015; Schuh et al., 2021), we further infer that UPB might be more helpful than OCB in terms of building a “good employee” image in the workplace, thus making the moderating effect of impression management motives on the relationship between pro-organizational motives and UPB stronger than that on the relationship between pro-organizational motives and OCB.

**FIGURE 1:**
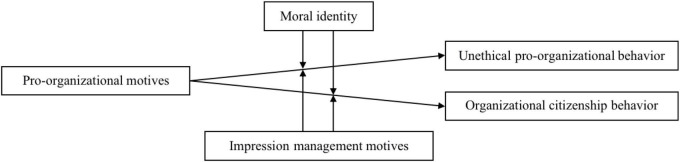
The research model.

In brief, this study is to explore the interactions of pro-organizational motives, moral identity, and impression management motives on UPB and OCB. The research model is depicted in Figure 1. In doing so, our study contributes to the literature in three ways. First, by taking both UPB and OCB into account and examining the common boundaries of the relationships between pro-organizational motives, UPB, and OCB, we not only provide accumulated evidence for some relationships that have been proved in prior research (e.g., the relationship between pro-organizational motives and OCB; Rioux and Penner, 2001) but also verify some new relationships (e.g., the interaction of impression management motives and pro-organizational motives on UPB), thereby providing a comprehensive explanation of the contingent relationships between pro-organizational motives and pro-organizational behaviors. Second and more specifically, our research enriches the knowledge about the interactions of multiple motives on pro-organizational behaviors (Bolino and Grant, 2016). On the one hand, by testing the interaction of pro-organizational motives and impression management motives on UPB, we respond to the call to adopt a motivational perspective to understand UPB (Cheng and Lin, 2019). On the other hand, to our knowledge, we are the first to generate evidence for the interaction of pro-organizational motives and impression management motives on OCB directed at the organization (Takeuchi et al., 2015). Third, we deepen the understanding of impression management motives by verifying the strength difference of the moderating effects of impression management motives on the relationships between pro-organizational motives and different pro-organizational behaviors. This finding shows that even when the moderating directions of impression management motives are the same, the moderating strength may still vary across the valence of the expected outcomes.

## Theory and hypotheses

### Pro-organizational motives and behaviors

Pro-organizational behaviors are employees’ acts that are neither specified in formal job descriptions nor ordered by leaders but are undertaken to benefit or help the organization (Brief and Motowidlo, 1986). According to the morality of the behaviors, pro-organizational behaviors can be classified into two different categories: ethical vs. unethical. UPB is unethical acts conducted by employees to potentially benefit the organization (Umphress et al., 2010). Examples of UPB include exaggerating the truth about the company’s products to customers, withholding negative information about the company from clients, and so on (Mishra et al., 2022). OCB is seen as the prototypical ethical pro-organizational behavior and can be differentiated into two types according to the beneficiary: OCB directed at individuals and OCB directed at the organization (Williams and Anderson, 1991). In this study, we focus on the latter type because it is more directly related to pro-organizational motives; meanwhile, for ease of presentation, OCB is used to refer to OCB directed at the organization in the following sections. Examples of OCB include showing pride when representing the organization in public, keeping up with developments in the organization, and so on (Lee and Allen, 2002).

When exploring the formation of behaviors, more and more researchers adopt the motivational perspective (Organ, 1990; Bolino, 1999; Borman and Penner, 2001; Cheng and Lin, 2019). The core premise of the motivational perspective is that to understand why certain behaviors emerge, it is vital to find out the reasons that guide the decision to conduct those behaviors (Katz, 1964; Penner et al., 1997). Extant research has widely demonstrated the predictive effects of pro-organizational motives on pro-organizational behaviors. For instance, Takeuchi et al. (2015) found that pro-organizational motives, which can also be termed organizational concern motives, had a significant and positive effect on OCB. A similar finding has also been shown in Rioux and Penner’s (2001) work. Dou et al. (2019) proposed and verified that the sense of belongingness motivated employees to assign more weight to the interests of their organization and then prompted them to engage in pro-organizational behaviors, even at the expense of moral standards. Hence, based on the above theoretical foundation and empirical evidence, we infer that employees with pro-organizational motives are likely to engage in pro-organizational behaviors and that the pro-organizational behaviors include UPB and OCB.

H1: Pro-organizational motives are positively related to UPB.

H2: Pro-organizational motives are positively related to OCB.

### The moderating role of moral identity

Although pro-organizational motives are likely to trigger pro-organizational behaviors (i.e., UPB and OCB), we argue that these relationships may vary between individuals, because different people have different assessments of the outcomes of UPB and OCB, which may result in decision differences. In this study, we focus on moral identity and impression management motives as the moderators of the relationships between pro-organizational motives, UPB, and OCB. Moral identity refers to “a self-conception organized around a set of moral traits” (Aquino and Reed, 2002, p. 1424) and reflects the extent to which a person sees being a moral person as central to his or her self-conception. We expect moral identity to weaken the effect of pro-organizational motives on UPB but strengthen the effect of pro-organizational motives on OCB, and we elaborate on this thought below.

As one kind of self-conception, moral identity involves a set of moral traits, such as compassion, honesty, friendliness, and so on (Aquino and Reed, 2002). Individuals with high moral identity place much importance on their behavioral morality and tend to act in ways that are consistent with what they deem a moral person should do; if not, they will experience dissonance and self-condemnation (Blasi, 1984). As noted at the outset, although UPB is a kind of pro-organizational behavior, UPB is also essentially unethical (Umphress et al., 2010). Apparently, a moral person should not undertake immoral behavior (e.g., UPB). If a moral person wants to contribute to the organization, engaging in ethical pro-organizational behavior (e.g., OCB) will be an optimal means. Hence, we infer that a high moral identity may encourage employees to express their pro-organizational motives in ethical forms of pro-organizational behavior (e.g., OCB) rather than unethical ones (e.g., UPB). That is, in the case of high moral identity, pro-organizational motives have a strong effect on OCB but a weak effect on UPB. In contrast, for individuals with low moral identity, their desire to keep the self-consistency between the real self and the ideal self is not strong (Aquino and Freeman, 2009). Thus, compared to employees with higher levels of moral identity, these employees are more likely to achieve their pro-organizational intent by unethical rather than ethical means (Matherne and Litchfield, 2012; Wang et al., 2019). Based on the above argument, we propose that moral identity may function as a moderator regulating the positive effects of pro-organizational motives on UPB and OCB.

H3: Moral identity moderates the relationship between pro-organizational motives and UPB such that the relationship is weaker for employees with high moral identity.

H4: Moral identity moderates the relationship between pro-organizational motives and OCB such that the relationship is stronger for employees with high moral identity.

### The moderating role of impression management motives

Apart from the moral identity, we propose that impression management motives, one’s desire to be seen positively and avoid being seen negatively (Rioux and Penner, 2001), may also serve as a boundary condition of the relationship between pro-organizational motives and pro-organizational behaviors. Specifically, we expect that impression management motives may strengthen the positive effects of pro-organizational motives on UPB and OCB, and we elaborate on this thought below.

According to Leary and Kowalski (1990), people with strong impression management motives pay much attention to bolstering their images and avoid creating an unfavorable self-representation in the eyes of others. For these people, behaviors that can help them look good are rather attractive. In the organizational context, actively engaging in pro-organizational behaviors is an effective way for employees to establish a “good solider” image, and a very typical example of such behaviors is OCB (Bolino, 1999). Given that conducting OCB helps employees create a favorable self-representation, we infer that compared to employees with weak impression management motives, employees with strong impression management motives are more likely to convert pro-organizational motives into pro-organizational behaviors (e.g., OCB). Partially in support of this argument, Grant and Mayer (2009) found in two studies that impression management motives strengthen the positive effect of prosocial motives on affiliative citizenship behaviors (e.g., showing courtesy to coworkers).

Will impression management motives also strengthen the positive effect of pro-organizational motives on UPB? To answer this question, we need to analyze whether undertaking UPB can help employees create a “good solider” image. As noted at the outset, UPB is harmful to organizational long-term performance, but may likely bring short-term benefits to the organization (Wang et al., 2020). Given that organizational long-term performance can be affected by many factors, people in the workplace may likely base their perceptions or judgments of a focal person on his or her short-term contributions to the organization. Meanwhile, because UPB is mainly directed at customers (Umphress et al., 2010), it is not easy for supervisors and coworkers to recognize the morality of the focal person’s behaviors. Taken together, engaging in UPB may improve actors’ contributions to the organization with low risks, thus helping actors build a favorable image. In line with the logic of the interaction of pro-organizational motives and impression management motives on OCB, we expect impression management motives to amplify the pro-organizational motives–UPB relationship.

H5: Impression management motives moderate the relationship between pro-organizational motives and UPB such that the relationship is stronger for employees with high impression management motives.

H6: Impression management motives moderate the relationship between pro-organizational motives and OCB such that the relationship is stronger for employees with high impression management motives.

Although we expect impression management motives to amplify the relationships between pro-organizational motives, UPB, and OCB, we further infer that the magnitude of the moderating effects of impression management motives may exist in subtle differences. Prior research suggests that ethical and legal expectations constrain the contributions a person can make to the organization in a given situation (Thau et al., 2015). In other words, unethical behavior often allows employees to contribute to the organization beyond what can be accomplished by moral means (Schuh et al., 2021). In this vein, we expect that for employees with strong impression management motives, UPB will be more attractive than OCB, thus making them more willing to convert pro-organizational motives into UPB than OCB. Accordingly, we infer that the moderating effect of impression management motives on the relationship between pro-organizational motives and UPB may be stronger than that on the relationship between pro-organizational motives and OCB.

H7: Compared to the relationship between pro-organizational motives and OCB, the relationship between pro-organizational motives and UPB is more strongly moderated by impression management motives.

## Methods

### Participants and procedures

Data were collected from an internet technology service company located in Chengdu, China. The main business of this company is to provide online advertisement and financial information services for the enterprises of service industries (e.g., catering). Participants were full-time staff working in the sales departments who need to frequently contact the clients. Before carrying out the survey, we presented to the HR director and the 252 participants the purpose, procedure, and confidentiality of our study and gained generous support. We took a two-stage questionnaire survey. To match the stage-one and stage-two questionnaires, we created the matching codes (i.e., three capital letters and a three-digit number) and hid them in the introduction part of the questionnaire (e.g., “Thanks very much for participating in this survey. This survey is to investigate…and is supported by…Foundation [Project No. BGL134]…”). Each matching code corresponded to one participant. In the first stage, the 252 participants were asked to rate their pro-organizational motives, moral identity, and impression management motives. After receiving all questionnaires, we checked them and filtered 29 invalid ones (e.g., all answers are the same), thereby obtaining 223 valid ones. About 1 month later, in the second stage, the 223 valid participants who attended the first-stage survey were invited to report their UPB and OCB. Again, after receiving all questionnaires, we checked them and deleted the invalid ones. The final valid sample consisted of 218 participants. Among the 218 participants, 48.165% were female; the average age was 29.615 years old (SD = 3.801); the average organizational tenure was 2.931 years (SD = 1.554).

### Measures

We followed the translation and back-translation procedure to translate the original English scales into Chinese. Unless otherwise indicated, all items were measured on 5-point Likert scales ranging from “1 = strongly disagree” to “5 = strongly agree.”

### Pro-organizational motives

The ten-item scale of organizational concern motives developed by Rioux and Penner (2001) was adopted to measure pro-organizational motives. A sample item is “Because I care what happens to the company.” The Cronbach’s α for this scale was 0.947.

### Moral identity

Moral identity was measured using the five-item scale of moral identity internalization developed by Aquino and Reed (2002). A sample item is “It would make me feel good to be a person who has these characteristics.” The Cronbach’s α for this scale was 0.819.

### Impression management motives

The ten-item scale of impression management motives developed by Rioux and Penner (2001) was adopted to evaluate impression management motives. A sample item is “Because I fear appearing irresponsible.” The Cronbach’s α for this scale was 0.808.

### Unethical pro-organizational behavior

UPB was measured with the six-item scale developed by Umphress et al. (2010). A sample item is “If it would help my organization, I would exaggerate the truth about my company’s products or services to customers and clients.” The Cronbach’s α for this scale was 0.785.

### Organizational citizenship behavior

The eight-item scale of OCB directed at the organization developed by Lee and Allen (2002) was adopted to measure OCB. A sample item is “Defend the organization when other employees criticize it.” The Cronbach’s α for this scale was 0.749.

### Control variables

Previous research suggests that some demographics (i.e., gender, age, and organizational tenure) may affect UPB and OCB (Matherne et al., 2018). Hence, we controlled for these variables. Specifically, gender was coded as a dummy variable (0 = female, 1 = male); age and organizational tenure were measured in the number of years.

### Analytic strategy

Using Mplus 8.3 and SPSS 26.0, we first conducted a series of confirmatory factor analyses to test the distinctiveness of our key variables and common method bias. Then, we reported the means and standard deviations of all variables and the correlations between them. To test hypotheses, we adopted hierarchical regression and simple slope analyses. Meanwhile, the bootstrapping approach was employed to estimate confidence intervals (CIs) at 95% significance (20,000 repetitions).

## Results

### Confirmatory factor analyses

Table 1 presents the confirmatory factor analyses results. According to Table 1, the five-factor model provided a better fit to the data (χ^2^ = 835.116, *df* = 692, χ^2^/*df* = 1.201, CFI = 0.959, TLI = 0.956, RMSEA = 0.031, SRMR = 0.052) than alternative models, thus verifying the distinctiveness of our measures. The single-factor model provided a poor fit (χ^2^ = 2291.480, *df* = 702, χ^2^/*df* = 3.264, CFI = 0.546, TLI = 0.521, RMSEA = 0.102, SRMR = 0.145), thereby indicating that common method bias was not a substantial issue in this study.

**TABLE 1:** The results of confirmatory factor analyses.

Model	χ ^2^	*df*	χ ^2^/*df*	χ ^2^ (*df*)	CFI	TLI	RMSEA	SRMR
Five-factor model: PM, MI, IMM, UPB, OCB	835.116	692	1.207	–	0.959	0.956	0.031	0.052
Four-factor model: PM, MI, IMM + UPB, OCB	1056.224	696	1.518	221.108***(4)	0.897	0.890	0.049	0.081
Three-factor model: PM, MI + OCB, IMM + UPB	1276.082	699	1.826	440.966***(7)	0.835	0.825	0.062	0.116
Two-factor model: PM + IMM + UPB, MI + OCB	1804.909	701	2.575	969.793***(9)	0.685	0.667	0.085	0.138
One-factor model: PM + MI + IMM + UPB + OCB	2291.480	702	3.264	1456.364***(10)	0.546	0.521	0.102	0.145

n = 218. PM, pro-organizational motives; MI, moral identity; IMM, impression management motives. + represents factors combined. ***p < 0.001.

### Descriptive statistics

Table 2 reports the means, SDs, and correlations of variables. As expected, pro-organizational motives were positively related to UPB (*r* = 0.463, *p* < 0.001) and OCB (*r* = 0.298, *p* < 0.001); moral identity was negatively related to UPB (*r* = −0.480, *p* < 0.001) but positively related to OCB (*r* = 0.557, *p* < 0.001); impression management motives were positively related to UPB (*r* = 0.561, *p* < 0.001) and OCB (*r* = 0.384, *p* < 0.001).

**TABLE 2:** Means, standard deviations, and correlations.

Variable	*M*	*SD*	1	2	3	4	5	6	7
1. Gender	0.482	0.501							
2. Age	29.615	3.801	–0.072						
3. Tenure	2.931	1.554	0.049	0.453***					
4. PM	3.529	0.854	–0.036	0.112	0.070				
5. MI	3.377	0.553	–0.086	–0.087	–0.064	−0.318***			
6. IMM	3.442	0.435	–0.017	–0.008	−0.139*	0.157*	–0.038		
7. UPB	3.127	0.457	0.017	–0.069	–0.077	0.463***	−0.480***	0.561***	
8. OCB	3.432	0.422	–0.110	0.001	–0.071	0.298***	0.557***	0.384***	–0.060

n = 218. PM, pro-organizational motives; MI, moral identity; IMM, impression management motives. *p < 0.05; ***p < 0.001.

### Hypotheses testing

We conducted hierarchical regression and simple slope analyses to test the hypotheses. At the same time, we used the bootstrapping approach to calculate 95% CIs. The results of hierarchical regression are displayed in Table 3. H1 and H2 predicted the positive links between pro-organizational motives and pro-organizational behaviors (i.e., UPB and OCB). According to Model 2 and Model 6 in Table 3, we found that pro-organizational motives had positive effects on UPB (*b* = 0.256, *p* < 0.001) and OCB (*b* = 0.149, *p* < 0.001). Hence, H1 and H2 were supported.

**TABLE 3:** The results of hierarchical regression analyses.

Variable	UPB				OCB			
		
	Model 1	Model 2	Model 3	Model 4	Model 5	Model 6	Model 7	Model 8
Intercept	3.127***	3.127***	3.109***	3.118***	3.432***	3.432***	3.450***	3.425***
Gender	0.015	0.029	–0.010	0.032	–0.087	–0.080	–0.015	–0.077
Age	–0.005	–0.010	–0.012	–0.012	0.003	–0.001	0.003	–0.001
Tenure	–0.018	–0.021	–0.027	0.004	–0.022	–0.024	–0.018	–0.008
PM		0.256***	0.221***	0.229***		0.149***	0.234***	0.136***
MI			−0.317***				0.553***	
IMM				0.530***				0.333***
PM × MI			−0.121*				0.115**	
PM × IMM				0.159**				0.135*
*R* ^2^	0.008	0.234	0.379	0.490	0.017	0.106	0.585	0.231
Δ*R*^2^	0.008	0.226***	0.145***	0.256***	0.017	0.089***	0.479***	0.125***

n = 218. PM, pro-organizational motives; MI, moral identity; IMM, impression management motives. *p < 0.05; **p < 0.01; ***p < 0.001.

**FIGURE 2:**
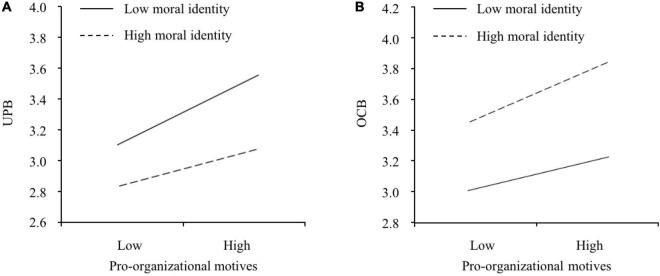
The moderating effects of moral identity on **(A)** the relationship between pro-organizational motives and UPB and **(B)** the relationship between pro-organizational motives and OCB.

H3 and H4 predicted the moderating role of moral identity. Model 3 revealed that moral identity weakened the relationship between pro-organizational motives and UPB (*b* = −0.121, *p* = 0.018). The simple slope test showed that this relationship was more positive when moral identity was low (one SD below the mean; *slope* = 0.288, 95% CI = [0.187, 0.385]) than high (one SD above the mean; *slope* = 0.154, 95% CI = [0.100, 0.209]) and that these two slopes significantly differed from each other (*slope difference* = −0.134, 95% CI = [−0.232, −0.036]). This interaction is plotted in Figure 2. Hence, H3 was supported. Model 7 showed that moral identity strengthened the relationship between pro-organizational motives and OCB (*b* = 0.115, *p* = 0.003). The simple slope test showed that this relationship was more positive when moral identity was high (one SD above the mean; *slope* = 0.298, 95% CI = [0.236, 0.359]) than low (one SD below the mean; *slope* = 0.171, 95% CI = [0.089, 0.268]) and these two slopes significantly differed from each other (*slope difference* = 0.127, 95% CI = [0.026, 0.218]). This interaction is plotted in Figure 2. Hence, H4 was supported.

**FIGURE 3:**
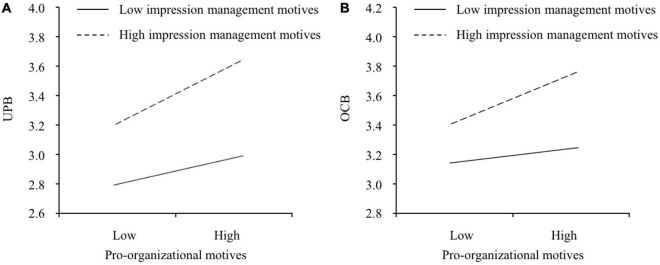
The moderating effects of impression management motives on **(A)** the relationship between pro-organizational motives and UPB and **(B)** the relationship between pro-organizational motives and OCB.

H5, H6, and H7 predicted the moderating effects of impression management motives. Model 4 revealed that impression management motives strengthened the link between pro-organizational motives and UPB (*b* = 0.159, *p* = 0.007). The simple slope test showed that this link was more positive when impression management motives were high (one SD above the mean; *slope* = 0.299, 95% CI = [0.208, 0.389]) than low (one SD below the mean; *slope* = 0.160, 95% CI = [0.103, 0.221]) and these two slopes significantly differed from each other (*slope difference* = 0.139, 95% CI = [0.036, 0.239]). This interaction is plotted in Figure 3. Hence, H5 was supported. Model 8 revealed that impression management motives strengthened the relationship between pro-organizational motives and OCB (*b* = 0.135, *p* = 0.042). The simple slope test showed that this relationship was more positive when impression management motives were high (one SD above the mean; *slope* = 0.194, 95% CI = [0.105, 0.291]) than low (one SD below the mean; *slope* = 0.077, 95% CI = [0.025, 0.139]) and these two slopes significantly differed from each other (*slope difference* = 0.117, 95% CI = [0.022, 0.214]). This interaction is plotted in Figure 3. Hence, H6 was supported. In addition, according to Model 4 and Model 8, we found that the moderating effect of impression management motives on the relationship between pro-organizational motives and UPB (*b* = 0.159, *p* = 0.007) was more significant than the moderating effect of impression management motives on the link between pro-organizational motives and OCB (*b* = 0.135, *p* = 0.042). Hence, H7 was supported.

## Discussion

Based on prior motivational research on UPB and OCB (Grant and Mayer, 2009; Takeuchi et al., 2015; Cheng and Lin, 2019), we developed a comprehensive model to explain the contingent relationships between pro-organizational motives and pro-organizational behaviors. We examined this model using time-lagged data from 218 Chinese salespeople. The results showed that pro-organizational motives were positively related to UPB and OCB. This finding is not surprising because it is consistent with the general notion that pro-organizational motives are critical drivers of pro-organizational behaviors (Rioux and Penner, 2001). We also found that moral identity served as a vital boundary of the effects of pro-organizational motives on UPB and OCB. That is, moral identity weakened the positive effect of pro-organizational motives on UPB but amplified the positive effect of pro-organizational motives on OCB. Moral identity captures the extent to which morality is important to one’s sense of identity (Matherne et al., 2018). Compared to employees with low moral identity, employees with high moral identity are more likely to recognize the unethical aspect of UPB. Accordingly, moral identity may guide employees to express their pro-organizational motives in ethical rather than unethical forms of pro-organizational behavior. Furthermore, we found that impression management motives amplified the positive effects of pro-organizational motives on UPB and OCB, and the interaction of pro-organizational and impression management motives was stronger on UPB than on OCB. Researchers have pointed out that engaging in pro-organizational behaviors can contribute to creating a favorable self-presentation (Jones and Pittman, 1982). If an employee has a strong desire to be seen positively (i.e., impression management motives), his or her pro-organizational motives may likely be easier to be converted into pro-organizational behaviors, especially the behaviors that have more valence.

### Theoretical implications

The present study makes several theoretical contributions. As noted at the outset, although scholars have explicitly or implicitly explored the boundaries of the impacts of pro-organizational motives on pro-organizational behaviors (e.g., Grant and Mayer, 2009; Matherne and Litchfield, 2012; Takeuchi et al., 2015; Wang et al., 2019), extant research is somewhat fragmented and thus in need of synthesis and extension. By investigating the moderating effects of moral identity and impression management motives on the relationships between pro-organizational motives, UPB, and OCB, this study narrows the aforementioned gap to some extent and provides a comprehensive and systematic explanation of the contingent relationships between pro-organizational motives and pro-organizational behaviors.

More specifically, we enrich the motivational research on UPB. In their critical review of UPB from a motivational perspective, Cheng and Lin (2019) suggested that apart from pro-organizational motives, self-serving motives can also affect the formation of UPB through the interaction with pro-organizational motives. Indirectly in support of this viewpoint, prior research has found that factors that may reflect one’s motives (e.g., organizational identification reflects one’s pro-organizational motives, and manipulative personality reflects one’s self-serving motives; Naseer et al., 2020) can interact to affect UPB. We go one step further. By examining the interaction of pro-organizational motives and impression management motives (one typical kind of self-serving motives) on UPB, we are among the first to generate direct evidence for the viewpoint of Cheng and Lin (2019).

Meanwhile, this study enriches the motivational research on OCB directed at the organization. Although prior studies have verified the direct effects of pro-organizational motives and impression management motives on OCB directed at the organization (Rioux and Penner, 2001; Takeuchi et al., 2015), the interaction of pro-organizational motives and impression management motives on such kind of OCB has not been demonstrated. Different from Takeuchi et al.’s (2015) unverified opinion that impression management motives may weaken the effect of pro-organizational motives on OCB directed at the organization, we deem that impression management motives may strengthen this link in some cases (e.g., the professions and organizations that emphasize personal striving and encourage individuals to be conspicuous). The data from salespeople in an internet technology service company supported our viewpoint, thereby deepening the current understanding of the formation of OCB.

Finally, this study contributes to the impression management motives literature. Earlier research has identified several impression management tactics (e.g., ingratiation, self-promotion, and so on; Jones and Pittman, 1982), suggesting that individuals with high impression management motives will have more willingness to engage in certain behaviors than others. That is, there exist differences in the magnitude of the direct impacts of impression management motives on individual behaviors. In comparison, few studies have paid attention to whether there also exist differences in the magnitude of the moderating effects of impression management motives. By demonstrating that the interaction of impression management motives and pro-organizational motives on UPB is a little stronger than that on OCB, we enrich the knowledge about impression management motives to some extent.

### Practical implications

This study has several implications for management. First, managers should have dialectical thinking and be aware that pro-organizational motives have the bright side and the dark side. If managers only realize the benefits of pro-organizational motives but do not recognize the risks of pro-organizational motives, their measures to enhance employees’ pro-organizational motives may likely cause hidden troubles for the long-term development of the organization. Second, to guide employees with strong pro-organizational motives to conduct more OCB and less UPB, an effective strategy is to enhance employees’ moral identity. On the one hand, when recruiting employees, managers should find out and hire candidates who have high moral identity. On the other hand, after employees have joined the organization, ethical training programs and other management practices that contribute to the improvement of employees’ moral identity are highly needed. Third, we found that the strengthening impact of impression management motives on the relationship between pro-organizational motives and UPB was stronger than that on the relationship between pro-organizational motives and OCB. This finding once again tells managers that dialectical thinking is a very important ability. Managers, especially those in organizations that highlight individual striving and encourage employees to be conspicuous, should know that impression management motives are a double-edged sword and may do more harm than good. Similar to Takeuchi et al. (2015), we may advise managers to discourage employees from making contributions when they appear to be doing so to improve their image.

### Limitations and future directions

This study has several limitations that need to be addressed in future research. The first limitation is related to the generalizability of our findings, as our data were collected only from one company. In fact, we do not deem that our findings can be generalized to all companies. As previously discussed, we think that similar findings may be obtained by collecting data from organizations that emphasize personal striving and encourage employees to be conspicuous. Nevertheless, the data collected from only one of these companies is difficult to reflect the representativeness of the sample data that we focus on. Hence, we advise future research to collect more data from these companies to retest our model. The second limitation concerns the research design. Although motives are often seen as the powerful drivers of behaviors, the time-legged research design is not able to rule out the possibility of reserve causality. To make the causality more clear, future research is strongly suggested to collect the data of UPB and OCB at Time 1 and control for them when examining the effects of pro-organizational motives on UPB and OCB at Time 2. Another potential limitation of this research is the use of self-report to assess individuals’ traits, motives, and behaviors, which may evoke concerns about common method bias and social desirability. We deem that self-reports are suitable to some extent, as others may not have the insight necessary to assess the focal person’s moral identity, pro-organizational motives, impression management motives, and UPB. To control common method bias, we followed Podsakoff et al.’s (2003) recommendations to adopt the temporal separation of measurement (one of the procedural remedies) and conduct the confirmatory factor analyses (one of the statistical remedies), the result of which showed that common method bias was not a serious threat in our study. Nevertheless, there still remains room for improvement. For instance, future research can collect data on OCB from observers. To reduce the effect of social desirability, we followed prior research (e.g., Takeuchi et al., 2015) to assure participants of the confidentiality of their responses. Future research can also address this issue methodologically by controlling for social desirability in the analyses.

## Conclusion

Finally, this study provides an integrative understanding of the contingent relationships between pro-organizational motives, UPB, and OCB by identifying moral identity and impression management motives as the boundaries and investigating their moderating effects. The findings not only contribute to the research on UPB, OCB, and impression management motives but also offer several suggestions that managers can follow to guide employees with pro-organizational motives to take moral behavior.

## Data availability statement

The data that support the findings of this study are available from the first author KC, chengken@zjut.edu.cn, upon reasonable request.

## Ethics statement

Ethical review and approval was not required for the study on human participants in accordance with the local legislation and institutional requirements. Written informed consent from the patients/participants or patients/participants legal guardian/next of kin was not required to participate in this study in accordance with the national legislation and the institutional requirements.

## Author contributions

KC and LG conducted conceptualization and data analysis and wrote the first draft of the manuscript. PH and YW performed the material preparation and data analysis. YL commented on previous versions of the manuscript. All authors contributed to the article and approved the submitted version.

## References

[B1] Aquino K. Freeman D. (2009). “Moral identity in business situations: a social-cognitive framework for understanding moral functioning,” in *Moral Personality, Identity, and Character*, eds Narvaez D. Lapsley D. K. (New York, NY: Cambridge University Press), 375–395.

[B2] Aquino K. Reed A. (2002). The self-importance of moral identity. *J. Pers. Soc. Psychol.* 83 1423–1440. 10.1037/0022-3514.83.6.1423 12500822

[B3] Baker B. Derfler-Rozin R. Pitesa M. Johnson M. (2019). Stock market responses to unethical behavior in organizations: an organizational context model. *Organizat. Sci.* 30 319–336. 10.1287/orsc.2018.1244 19642375

[B4] Bennis W. M. Medin D. L. Bartels D. M. (2010). The costs and benefits of calculation and moral rules. *Perspect. Psychol. Sci.* 5 187–202. 10.1177/1745691610362354 26162125

[B5] Blasi A. (1984). “Moral identity: its role in moral functioning,” in *Morality, Moral Behavior, and Moral Development*, eds Kurtines W. Gewirtz J. (New York, NY: Wiley), 128–139.

[B6] Bolino M. C. (1999). Citizenship and impression management: good soldiers or good actors? *Acad. Manag. Rev.* 24 82–98.

[B7] Bolino M. C. Grant A. M. (2016). The bright side of being prosocial at work, and the dark side, too: a review and agenda for research on other-oriented motives, behavior, and impact in organizations. *Acad. Manag. Ann.* 10 599–670. 10.5465/19416520.2016.1153260

[B8] Borman W. C. Penner L. A. (2001). “Citizenship performance: its nature, antecedents, and motives,” in *Personality Psychology in the Workplace*, eds Roberts B. W. Hogan R. (Washington, DC: American Psychological Association), 45–61.

[B9] Brief A. P. Motowidlo S. J. (1986). Prosocial organizational behavior. *Acad. Manag. Rev.* 11 710–725. 10.2307/258391

[B10] Chen M. Chen C. C. Sheldon O. J. (2016). Relaxing moral reasoning to win: how organizational identification relates to unethical pro-organizational behavior. *J. Appl. Psychol.* 101 1082–1096. 10.1037/apl0000111 27100068

[B11] Cheng K. Lin Y. (2019). Unethical pro-organizational behavior: a motivational perspective. *Adv. Psychol. Sci.* 27 1111–1122. 10.3724/SP.J.1042.2019.01111 34658988

[B12] Dou K. Chen Y. Liu J. Li J. Wang Y. (2019). Why and when does job satisfaction promote unethical pro-organizational behaviours? Testing a moderated mediation model. *Int. J. Psychol.* 54 766–774. 10.1002/ijop.12528 30238509

[B13] Grant A. M. Mayer D. M. (2009). Good soldiers and good actors: prosocial and impression management motives as interactive predictors of affiliative citizenship behaviors. *J. Appl. Psychol.* 94 900–912. 10.1037/a0013770 19594233

[B14] Jones E. E. Pittman T. S. (1982). “Toward a general theory of strategic self-presentation,” in *Psychological Perspectives on the Self*, ed. Suls J. (Hillsdale, NJ: Lawrence Erlbaum), 231–262.

[B15] Katz D. (1964). The motivational basis of organizational behavior. *Behav. Sci.* 9 131–146. 10.1002/bs.3830090206 5888769

[B16] Leary M. R. Kowalski R. M. (1990). Impression management: a literature review and two-component model. *Psychol. Bull.* 107 34–47. 10.1037/0033-2909.107.1.34

[B17] Lee K. Allen N. J. (2002). Organizational citizenship behavior and workplace deviance: the role of affect and cognitions. *J. Appl. Psychol.* 87 131–142. 10.1037/0021-9010.87.1.131 11916207

[B18] Matherne C. F. Litchfield S. R. (2012). Investigating the relationship between affective commitment and unethical pro-organizational behaviors: the role of moral identity. *J. Leadersh. Account. Ethics* 9 35–47.

[B19] Matherne C. F. Ring J. K. Farmer S. (2018). Organizational moral identity centrality: relationships with citizenship behaviors and unethical prosocial behaviors. *J. Bus. Psychol.* 33 711–726. 10.1007/s10869-017-9519-4

[B20] Mishra M. Ghosh K. Sharma D. (2022). Unethical pro-organizational behavior: a systematic review and future research agenda. *J. Bus. Ethics* 179 63–87. 10.1007/s10551-021-04764-w

[B21] Moorman R. H. Blakely G. L. Niehoff B. P. (1998). Does perceived organizational support mediate the relationship between procedural justice and organizational citizenship behavior. *Acad. Manag. J.* 41 351–357. 10.2307/256913 11302227

[B22] Naseer S. Bouckenooghe D. Syed F. Khan A. K. Qazi S. (2020). The malevolent side of organizational identification: unraveling the impact of psychological entitlement and manipulative personality on unethical work behaviors. *J. Bus. Psychol.* 35 333–346. 10.1007/s10869-019-09623-0

[B23] Organ D. W. (1988). *Organizational Citizenship Behavior: The Good Soldier Syndrome.* Lexington, MA: Lexington Books.

[B24] Organ D. W. (1990). “The motivational basis of organizational citizenship behavior,” in *Research in Organizational Behavior*, eds Staw B. M. Cummings L. L. (Greenwich, CT: JAI Press), 43–72.

[B25] Penner L. A. Midili A. R. Kegelmeyer J. (1997). Beyond job attitudes: a personality and social psychology perspective on the causes of organizational citizenship behavior. *Hum. Perform.* 10 111–132. 10.1207/s15327043hup1002_4

[B26] Podsakoff P. M. Mackenzie S. B. Lee J. Y. Podsakoff N. P. (2003). Common method biases in behavioral research: a critical review of the literature and recommended remedies. *J. Appl. Psychol.* 88 879–903. 10.1037/0021-9010.88.5.879 14516251

[B27] Podsakoff P. M. MacKenzie S. B. Paine J. B. Bachrach D. G. (2000). Organizational citizenship behaviors: a critical review of the theoretical and empirical literature and suggestions for future research. *J. Manag.* 26 513–563. 10.1177/014920630002600307

[B28] Rioux S. M. Penner L. A. (2001). The causes of organizational citizenship behavior: a motivational analysis. *J. Appl. Psychol.* 86 1306–1314. 10.1037/0021-9010.86.6.1306 11768072

[B29] Schuh S. C. Cai Y. Kaluza A. J. Steffens N. K. David E. M. Haslam A. (2021). Do leaders condone unethical pro-organizational employee behaviors? The complex interplay between leader organizational identification and moral disengagement. *Hum. Resour. Manag.* 60 969–989. 10.1002/hrm.22060

[B30] Takeuchi R. Bolino M. C. Lin C. C. (2015). Too many motives? The interactive effects of multiple motives on organizational citizenship behavior. *J. Appl. Psychol.* 100 1239–1248. 10.1037/apl0000001 25198096

[B31] Thau S. Derfler-Rozin R. Pitesa M. Mitchell M. S. Pillutla M. M. (2015). Unethical for the sake of the group: risk of social exclusion and pro-group unethical behavior. *J. Appl. Psychol.* 100 98–113. 10.1037/a0036708 24773402

[B32] Umphress E. E. Bingham J. B. (2011). When employees do bad things for good reasons: examining unethical pro-organizational behaviors. *Organ. Sci.* 22 621–640. 10.1287/orsc.1100.0559 19642375

[B33] Umphress E. E. Bingham J. B. Mitchell M. S. (2010). Unethical behavior in the name of the company: the moderating effect of organizational identification and positive reciprocity beliefs influencing unethical pro-organizational behavior. *J. Appl. Psychol.* 95 769–780. 10.1037/a0019214 20604596

[B34] Van Dick R. Grojean M. W. Christ O. Wieseke J. (2006). Identity and the extra mile: relationships between organizational identification and organizational citizenship behavior. *Br. J. Manag.* 17 283–301. 10.1111/j.1467-8551.2006.00520.x 14672147

[B35] Wang T. Long L. Zhang Y. He W. (2019). A social exchange perspective of employee–organization relationships and employee unethical pro-organizational behavior: the moderating role of individual moral identity. *J. Bus. Ethics* 159 473–489. 10.1007/s10551-018-3782-9

[B36] Wang T. Zhang Y. Zhou H. Zhang J. (2020). The negative effects and underlying mechanisms of unethical pro-organizational behavior. *Adv. Psychol. Sci.* 28 1246–1255. 10.3724/SP.J.1042.2020.01246

[B37] Williams L. J. Anderson S. E. (1991). Job satisfaction and organizational commitment as predictors of organizational citizenship and in-role behavior. *J. Manag.* 17 601–617. 10.1177/014920639101700305 18674448

